# Hydrogel Alginate Seed Coating as an Innovative Method for Delivering Nutrients at the Early Stages of Plant Growth

**DOI:** 10.3390/polym13234233

**Published:** 2021-12-02

**Authors:** Dawid Skrzypczak, Łukasz Jarzembowski, Grzegorz Izydorczyk, Katarzyna Mikula, Viktoria Hoppe, Karolina Anna Mielko, Natalia Pudełko-Malik, Piotr Młynarz, Katarzyna Chojnacka, Anna Witek-Krowiak

**Affiliations:** 1 Department of Advanced Material Technologies, Wroclaw University of Science and Technology, 50-370 Wroclaw, Poland; 246475@student.pwr.edu.pl (Ł.J.); grzegorz.izydorczyk@pwr.edu.pl (G.I.); katarzyna.mikula@pwr.edu.pl (K.M.); katarzyna.chojnacka@pwr.edu.pl (K.C.); anna.witek@pwr.edu.pl (A.W.-K.); 2 Center for Advanced Manufacturing Technologies (CAMT), Faculty of Mechanical Engineering, Wroclaw University of Science and Technology, Łukasiewicza 5, 50-371 Wrocław, Poland; viktoria.hoppe@pwr.edu.pl; 3 Department of Biochemistry, Molecular Biology and Biotechnology, Faculty of Chemistry, Wrocław University of Science and Technology, Łukasiewicza 2, 50-371 Wrocław, Poland; karolina.mielko@pwr.edu.pl (K.A.M.); natalia.pudelko-malik@pwr.edu.pl (N.P.-M.); piotr.mlynarz@pwr.edu.pl (P.M.)

**Keywords:** seed coating, macronutrients, micronutrients, alginate, biostimulant, alkaline hydrolysis

## Abstract

Seed coating containing fertilizer nutrients and plant growth biostimulants is an innovative technique for precision agriculture. Nutrient delivery can also be conducted through multilayer seed coating. For this purpose, sodium alginate with NPK, which was selected in a preliminary selection study, crosslinked with divalent ions (Cu(II), Mn(II), Zn(II)) as a source of fertilizer micronutrients, was used to produce seed coating. The seeds were additionally coated with a solution containing amino acids derived from high-protein material. Amino acids can be obtained by alkaline hydrolysis of mealworm larvae (Gly 71.2 ± 0.6 mM, Glu 55.8 ± 1.3 mM, Pro 48.8 ± 1.5 mM, Ser 31.4 ± 1.5 mM). The formulations were applied in different doses per 100 g of seeds: 35 mL, 70 mL, 105 mL, and 140 mL. SEM-EDX surface analysis showed that 70 mL of formulation/100 g of seeds formed a continuity of coatings but did not result in a uniform distribution of components on the surface. Extraction tests proved simultaneous low leaching of nutrients into water (max. 10%), showing a slow release pattern. There occurred high bioavailability of fertilizer nutrients (even up to 100%). Pot tests on cucumbers (*Cornichon de Paris*) confirmed the new method’s effectiveness, yielding a 50% higher fresh sprout weight and four times greater root length than uncoated seeds. Seed coating with hydrogel has a high potential for commercial application, stimulating the early growth of plants and thus leading to higher crop yields.

## 1. Introduction

Micronutrient deficiencies result in poor human health. It is estimated that the global population growth will require an increase in agricultural production of up to 50% in the next 30 years [1]. Multi-nutrient precise fertilization in the vicinity of plants could compensate for micronutrient deficiencies in them. Traditional preparations bring about the poor quality of air (NO_x_ emissions), water (leaching of micro- and macronutrients), and soil (accumulation of elements). Conventional applications require fertilization of whole crop areas, so only a small part of the product reaches the plant. Plants—especially at the beginning of growth when the root system is not formed yet—often do not have the required amounts of nutrients because of their uneven distribution. Seeds coated with nutrients could provide the plant with all it needs right after germination. It is essential to ensure proper development at this stage, contributing to higher yields in the future [2].

Coats around seeds contain compounds designed to directly deliver essential substances (symbiotic microorganisms, micronutrients, growth regulators) and are stuffed with protective chemicals (pesticides, herbicides, fungicides, insecticides, nematicides). This solution makes spraying or fertilizing redundant. Such precise application substantially reduces the doses of nutrients, so little is lost to the environment. Coated seeds can also be stored for a long time without losing their properties [1]. Such seeds are prepared by soaking them in solutions containing the desired compounds (e.g., micronutrients) and then drying them (or directly subjecting them to germination). Materials used for coating allow the adhesion of chemical or biological compounds to be introduced to the seed surface. The simplest solution is to coat finely ground solids or liquids dissolved in a solvent. The carrier can be an adhesive (arabic gum, xanthan gum), polysaccharide (alginate, chitosan), polymer (cellulose derivatives), and filler (peat, lime, biochar) [3]. The market for seed pelleting materials is estimated to grow extensively [4].

Seed coat designing has its requirements. Coats should be (i) nontoxic to seeds and environmentally friendly, (ii) biodegradable because the rate of degradation is a crucial parameter for the release of certain compounds, and (iii) of appropriate thickness because the rate of compound release and germination potential depends on it. The resultant selection of materials leads to the application of various biopolymers, including alginate-based compositions. Alginate is a popular material for preparing hydrogel formulations with slow/controll release properties. Alginate is a natural polysaccharide extracted from widely abundant brown algae. It can also be obtained by biosynthesis [5]. It is a linear polymer composed of β-D-mannuronic acid (M) and α-L-guluronic acid (G) subunits that crosslink in the presence of divalent cations to form stable gels. The material is nontoxic and biodegradable, easy to prepare, and has the ability to respond to environmental conditions (including pH) [6]. Hence, it is utilized in many industries, including medical, pharmaceutical, food, packaging, etc. Agricultural applications of alginate are also known, mainly as a carrier of fertilizer nutrients [7,8] or as a component of slow-release coatings [9]. Even though the use of this polysaccharide in seed applications is not fully explored, seeds are known to have been encapsulated in growth regulators in an alginate coating [10], bacteria, i.e., *Pseudomonas aeruginosa* LY-11 [11], and mycoparasites *Trichoderma asperellum* [12].

We developed a method for the delivery of macro- and micronutrients and biostimulants at early stages of plant growth through biodegradable seed coats and a method of hydrolysis of proteins of raw proteins materials of biological origin to free amino acids. Then, we analyzed the effects of individual coating thickness on seed structure and nutrient distribution on the surface. Extraction (in vitro) tests and in pot (in vivo) trials confirmed the controlled release of components and biostimulatory properties of plant growth.

To the best of our knowledge, this is the first work describing the preparation of coatings based on alginate solution containing macro- and micronutrients and an additional coating with biostimulation properties. Our manuscript follows the journal’s scope and addressed the total environment, specifically the biosphere, hydrosphere, and lithosphere. The study described the effect of novel nutrient-containing coatings on plant nutrition (biosphere). Thanks to their properties—low leaching, high bioavailability of nutrients—the coatings protect against excessive loss of macro- and microelements to water (hydrosphere) and soil (lithosphere), thus eliminating their contamination.

## 2. Materials and Methods

### 2.1. Materials

Sodium alginate (ALG), carboxymethyl cellulose (CMC), and gum arabic (GA) were purchased from Sigma Aldrich. POCH (Poznan, Poland) supplied the salts used to prepare the micronutrient solution, copper sulfate (CuSO_4_), zinc sulfate (ZnSO_4_), and manganese sulfate (MnSO_4_). The solution of macroelements was prepared from potassium chloride (KCl) and ammonium nitrate (NH_4_NO_3_) purchased from Sigma Aldrich and mono-ammonium phosphate ((NH₄)H₂PO₄) from PROTON (Jastrzebie-Zdroj, Poland). Potassium hydroxide (KOH) and orthophosphoric acid (H₃PO₄) used to prepare the biostimulant (AA) were from POCH (Poznan, Poland). Cucumber seeds (*Cornichon de Paris*) were from Legutko (Jutrosin, Poland).

### 2.2. Preparation of Seed Coatings

Seed coatings were prepared by two methods: dipping (Section 2.2.3) and spraying (Section 2.2.4).

#### 2.2.1. Preparation of NPK Solution

Solutions of alginate (ALG), gum arabic (GA), and carboxymethyl cellulose (CMC) were based on the NPK solution, which was prepared from mono-ammonium phosphate (MAP), potassium chloride (KCl), and ammonium nitrate (NH_4_NO_3_). The contents of each component were (*m/m*): 1.02% N, 3.32% P_2_O_5_, and 2.60% K_2_O.

#### 2.2.2. Biostimulant Preparation

The biostimulant (AA) with amino acids was prepared by alkaline hydrolysis of mealworm larvae using potassium hydroxide (KOH) solution (20%). Larvae, 50 g, were placed in a beaker and soaked in the solution. The mixture was incubated for 3 h at 80 °C. After this time, the hydrolysate was neutralized with ortho-phosphoric acid (28%) to pH = 5.15. Subsequently, the hydrolysate was centrifuged and the liquid phase was used for further studies. The clarified hydrolysate was stored at 4 °C. The separated solid phase can potentially be a material for the production of granular fertilizers.

#### 2.2.3. Coating by Dip Method

Natural polymers solution with different concentrations were prepared on the basis of the NPK solution (Table 1). For this purpose, 200 mL of NPK solution was measured and then the given polymer was dissolved. Seeds were coated by the dip method. Cucumber seeds (*Cornichon de Paris*) were placed in the prepared solutions for 10 min at room temperature. Subsequently, the material was drained and dried at a temperature not exceeding 40 °C. The dried seeds were sprayed with a portion of AA.

#### 2.2.4. Coating by Spray Method

Alginate solution (2% *m/m*) was selected for seed coating. The formulation was prepared by dissolving sodium alginate in NPK solution (see Section 2.2.1). Seeds were coated with 2% sodium alginate solution with NPK and crosslinked with a micronutrient solution prepared from sulfate salts (5000 mg/L Cu, 5000 mg/L Zn, 5000 mg/L Mn). The spray was applied at different doses (Table 2). The seeds were dried at a temperature not exceeding 40 °C. The dry material was additionally coated with varying portions of AA (37.5 mL/100 g; 75 mL/100 g; 112.5 mL/100 g; 150 mL/100 g) and dried again.

### 2.3. Characteristics of Coated Seeds

The coated seeds prepared by both methods were analyzed for physicochemical characterization, including surface analysis, extraction tests in water and ammonium citrate, and the performance of the coatings was verified in germination tests and pot trials.

#### 2.3.1. Surface Characterization

The structure of the coated seeds was observed under an optical microscope (LEICA DMi8, Wetzlar, Germany) using zoom × 2.5. Scanning electron microscopy (SEM) imaging technique was used to analyze the seeds’ morphology after various modifications described in this paper. Studies were performed with a Sigma VP-600 microscope (Zeiss, Jena, Germany), using the in LENS imaging mode. For high-resolution images, the samples attached to a conductive carbon tape on a dedicated to microscopic observations tables were covered with a conductive gold layer using a Q150R ES vacuum sputtering machine (Quorum Technologies, Lewes, UK). Chemical microanalysis was carried out from representative areas of the sample with an area of approximately 62,500 mm^2^ with an accelerating voltage of 20 kV, and data were collected by an EDAX Octane Elect EDS System detector (AMETEK, Berwyn, PA, USA).

#### 2.3.2. The Extraction Tests

Extraction tests were performed according to EN 15958:2011 (extraction in water) and EN 15957:2011 (extraction in neutral ammonium citrate) for seeds prepared in Section 2.2. 

To evaluate the leachability of the components in water, seeds (2.5 g) were weighed in an Erlenmeyer flask for water extraction and then 225 mL of distilled water was added. The prepared mixture of seeds soaked in water was transferred from flasks to a shaker and shaken for 30 min at room temperature. The solution was the separated from the seeds by filtration through a filter paper, and the filtrate was subjected to elemental composition analysis.

For extraction in ammonium citrate, 1 g of the tested coated seeds was weighed and then transferred to an Erlenmeyer flask containing 100 mL of neutral ammonium citrate heated to 65 °C. The flasks were placed on a shaker equipped with a heating chamber to maintain a constant temperature of 65 °C during shaking (1 h). The solutions were cooled and filled up to 250 mL with water, and then filtered before the analysis.

### 2.4. Germination Tests

Germination tests were conducted for ten days at 25 °C with uniform illumination of 2400 lux. Dedicated dishes and caps were used for the experiment. Cucumber (*Cornichon de Paris*) was selected as the test plant. Twenty-five seeds were placed equally spaced on glass Petri dishes lined with tissue paper (5 × 5 to occupy the entire surface). The light-protected dishes were stratified for two days (4 °C). The dishes were covered with caps to ensure constant humidity and moved under a light source (cycle day:night 16:8). The moisture content of the substrate was continuously monitored during germination. During the first days of the process, the effect of the thickening agent on the germination rate of the seeds was analyzed. After 10 days, the quality of plants was evaluated (root parameters, stem length, fresh weight of sprouts, and chlorophyll content). Chlorophyll content was analyzed using an OPTI-SCIENCES CCM-300 chlorophyllmeter (Hudson, NH, USA). Root ball analysis was performed using an Epson PerfectionV850 Pro camera (Nagano, Japan). Statistica 13 software (Statsoft, Australia, Turkey’s RIR test) was used to elaborate results statistically. Germination tests allowed the selection of a thickening agent that did not adversely affect plant growth stimulation.

### 2.5. Pot Trials

The pot trials were pre-tests for the field trials. Dedicated multiplexes (5 × 4) were used for the experiment. Pot trials were conducted for 20 days at 25 °C, with uniform illumination of 2400 lux, which was switched on a 16:8 cycle, 16 h of the day and 8 h of the night, respectively. Coated cucumber seeds (*Cornichon de Paris*, Legutko) were used as tested plants. Seeds without coating were a control group. Tests were performed on universal soil of pH = 5.5–6.5 containing NPK (14-16-18): 0.6 kg/m^3^ and salinity 1.9 g NaCl/dm^3^. Soil moisture was maintained at 60% during the tests. After 20 days, plant growth parameters (root parameters, stem length, fresh sprout weight, and chlorophyll content) were evaluated. Statistica software (Statsoft, Australia, Turkey’s RIR test) were used to elaborate the results statistically processed. Dried plants were subjected to mineralization and multi-element composition analysis.

### 2.6. Analytical Methods

#### 2.6.1. Macro- and Micronutrient Content Analysis

Prior to the analyses of macro- and micronutrient content in biological materials, samples were mineralized. The liquefaction of samples was performed in a microwave oven (Start D, Milestone, Fremont, CA, USA). The sample (0.5 g) was placed in a Teflon vessel and digested with spectrally pure nitric acid(V) (5 mL). The sealed samples were mineralized for 35 min at 200 °C (100–1000 W power).

Multielemental composition analysis was performed at the Accredited Chemical Laboratory for Multielemental Analysis at the Wrocław University of Science and Technology (PCA No. AB 696; ILAC/MRA) according to ISO 17025:2018. ICP-OES analyses were performed on Vista-MPX spectrometer (Varian, Palo Alto, CA, USA) [13].

#### 2.6.2. Analysis of the Amino Acid Composition of the Biostimulant

Neutralized samples were used for analysis (larvae hydrolyzate). The analysis was performed on triplicate replicates for each sample. A sample of hydrolysate (900 µL) of the sample was centrifuged at 14,000 rpm at 4 °C for 15 min. Further, supernatants were taken to a new Eppendorf type tube and mixed sequentially with 80 µL of D_2_O (ARMAR Chemicals, Döttingen, Switzerland) and 30 µl of TSP (ARMAR Chemicals, Döttingen, Switzerland) (20 mM) for a final 1 mM TSP concentration in 600 µL of the analyzed samples. Samples of ^1^H NMR spectra were acquired immediately after preparation.

#### 2.6.3. The ^1^H NMR Measurements and Concentration Measurement

Measured ^1^H NMR spectra of the hydrolysate samples were acquired at 298 K by means of an Advance II spectrometer (Bruker, GmBH, Bremen, Germany) operating at a proton frequency of 600.58 MHz. Spectra were measured with 1D NOESY (noesypr1d in Bruker notation) pulse sequence with water presaturation. The pulse sequence parameters were as follows: relaxation delay, RD = 4.0 s, acquisition time = 1.36 s, the total number of scan = 128, mixing time = 125 ms, time-domain data points = 32.768, receiver gain = 36. The spectra were processed with a line broadening of 0.3 Hz and were manually phased with Topspin 3.2 software (Bruker, GmBH, Germany). Hydrolysate samples spectra were referenced to the TSP signal and baseline corrected in MestReNova software (Mnova NMR ver. 14.1.1, Mestrelab Research, SOFTBOOKS S.C, Kraków, Poland). The amino acids resonances signals identification and concentration were obtained according to assignments and calculations in Chenomx software (Chenomx NMR Suite 8.5, Chenomx Inc., Edmonton, AB, Canada).

## 3. Results

### 3.1. An Initial Selection of Coating Materials

#### 3.1.1. Analysis of the Amino Acid Composition of the Biostimulant

Mealworm larvae are a high-protein material (54% crude protein on a dry weight basis). This material is becoming popular as an alternative food source due to the ease of cultivation [14]. The larvae contain nitrogen present in the form of protein, and subjecting them to a hydrolysis process makes it possible to obtain amino acids and short peptides [15]. Hydrolyzed mealworm larvae were analyzed for amino acid concentration. The total amino acid content was about 3% (*m/m*), indicating that the protein was not fully hydrolyzed to amino acid forms. Thus, short peptide chains were also present in the formed hydrolysate. Peptides are responsible for transmitting signals between the cells in the plant during growth and stress conditions [16].

**Figure 1:**
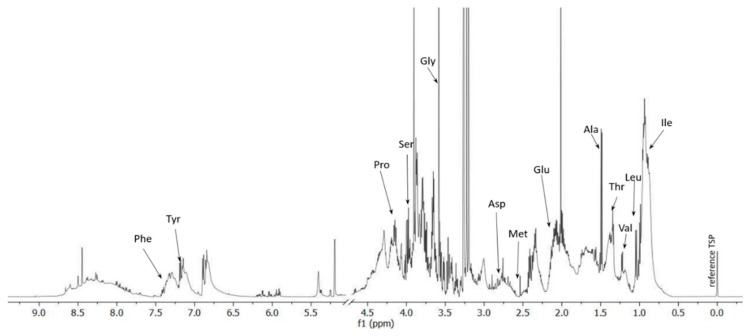
1D ^1^H NMR spectrum of the larvae hydrolysate.

Protein amino acids are a well-assimilable source of nitrogen and carbon, and they are ready building blocks for plants because they can be taken up by the root system and included in the metabolic pathways. Thanks to the presence of carboxyl and amino groups, they also act as natural chelating agents for cationic and anionic micronutrient ions, ensuring their high bioavailability [17]. Thirteen amino acids were identified in the hydrolyzed larvae sample: Gly, Ala, Val, Leu, Ile, Ser, Thr, Met, Pro, Phe, Tyr, Asp, and Glu (Table 3). The ^1^H NMR spectrum is presented in Figure 1. The highest concentrations were determined for glycine (71.2 mM), glutamate (55.8), proline (48.8), and seronine (31.4). Glycine is a component of plant tissues and chlorophyll. It assists photosynthesis by promoting the production of assimilates [18]. Proline reduces stress caused by the presence of heavy metals and high salinity [19,20]. In addition, proline is an osmolyte that prevents nutrient deficiency under high stress conditions (drought). Cacefo et al. (2021) confirmed that proline acts directly or indirectly as a mitigating factor for nutrient deficiencies caused by water deficit in tobacco. They concluded that proline protects against abiotic stress [21]. A similar effect can also be attributed to serine, which is also responsible for water management and anti-stress responses [22]. Additionally, this amino acid plays a significant role in glycolysis, Krebs cycle, or enzyme synthesis [23]. Glutamate promotes the uptake of both macro- and micro-nutrients, which may result in increased nutrient transfer: coating—plant [24].

In addition to amino acids and short peptides, oligo- and monosaccharides derived from the chitinous larval skin may have been present in the hydrolysate. These substances may also have beneficial effects on plant growth (e.g., enhancement of seed germination and photosynthesis, better nitrogen fixation) [25]. The application of the coating directly on the seeds can therefore have an AA effect on the growth of the above- and below-ground parts of the plant and strengthen plants under stress conditions.

#### 3.1.2. Effect of a Thickening Agent—Germination Tests

Seed coating based on/with biodegradable polymers enriched with macro- and micronutrients and amino acids appears to be a promising approach to precise plants breeding. The germination tests are preliminary studies carried out on a laboratory scale. In most of the available literature, this type of test is used to evaluate the material’s suitability as a fertilizer formulation [26]. The absence of phytotoxic effect (no negative impact of the preparation on the biometric parameters of plants) indicates that the material could be applied for seed coating. Selection of a suitable polymer that will not inhibit the first stages of plant growth and will be stable in time is—apart from testing of fertilizer materials—an essential element in preparing seed coats [12].

Germination tests checked the influence of new coatings based on sodium alginate, arabic gum, carboxymethylcellulose and additional AA coating on plant growth. The analysis of seed germination during the first three days of the study revealed a blocking effect of arabic gum on the first phase of plant growth (Table 4). No negative effect was seen in the group where alginate at a 2% concentration was applied. The additional AA-based coating slowed down germination due to the requirement for the plant to overcome the additional coating layer.

On day 10 of the study, a biometric evaluation of cucumber sprouts was performed, excluding groups of seeds with gum arabic coating. The measured parameters such as mean sprout weight, mean stem length, root ball parameters, and chlorophyll content for the coated seeds showed equal or higher values than those for uncoated seeds (Table 5).

In almost all cases, polymer coatings reduced the average stem length (S2, S3, S4) by about 30%, although these results were not always statistically significant. This is due to the delayed disruption of the plant through the polymer film. The application of AA (S5) delayed germination, but it also stimulated the growth of the aboveground parts of the cucumber. A positive effect of polymer films was found on root ball parameters (S2, S3, S5), especially for the group additionally coated with AA (S5), where root ball volume increased by about 40% compared with the control group. There was no statistically significant effect of thickening agents on the chlorophyll content. According to the determined parameters, one polymer was selected for further testing, ALG 2%, which did not show a phytotoxic effect, as also confirmed in other studies [27,28]. The stimulatory effect of amino acids derived from larvae was also tentatively confirmed in the tests, which qualified this material for further studies.

### 3.2. Effect of Coating Thickness on Application Properties

#### 3.2.1. Characteristics of Seeds Coating

##### Surface Characteristics

**Figure 2:**
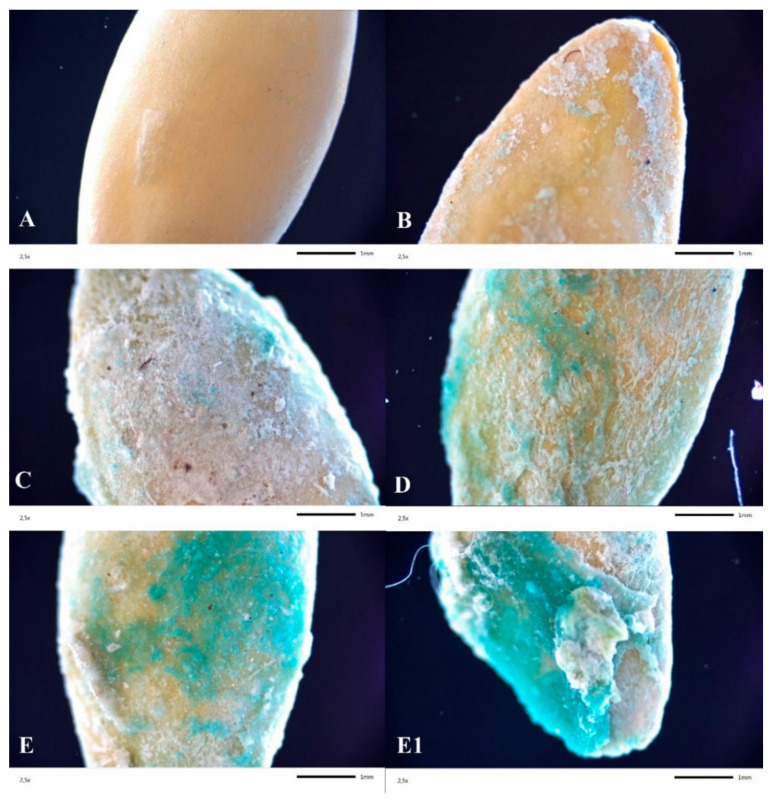
Optical microscope images of S1 (**A**), S5.1 (**B**), S5.2 (**C**), S5.3 (**D**) and S5.4 (**E**,**E1**), zoom ×2.5 (S1—Uncoated seeds, S5.1—Seed coating containing a solution of 2% alginate with amino acids (37.6 mL/100 g), micronutrients (37.5 mL/100 g), and NPK, S5.2—Seed coating containing a solution of 2% alginate with amino acids (75.1 mL/100 g), micronutrients (75.1 mL/100 g), and NPK, S5.3—Seed coating containing a solution of 2% alginate with amino acids (112 mL/100 g), micronutrients (112 mL/100 g), and NPK, S5.4—Seed coating containing a solution of 2% alginate with amino acids (150 mL/100 g), micronutrients (150 mL/100 g), and NPK).

Figure 2 shows applied seed coatings with different amounts of a crosslinking solution (Cu(II), Mn(II), Zn(II)) and an AA solution. A single dose of M and AA did not allow for uniform surface coverage (Figure 2B). The larger the number of applications, the more improved was the structural continuity. The triple coverage of the seed resulted in the crystallization of micronutrient salts on the surface (Figure 2E1). The microscopic image showed an uneven distribution of micro- and macronutrients across the seed surface, confirmed by SEM-EDAX analysis.

**Figure 3:**
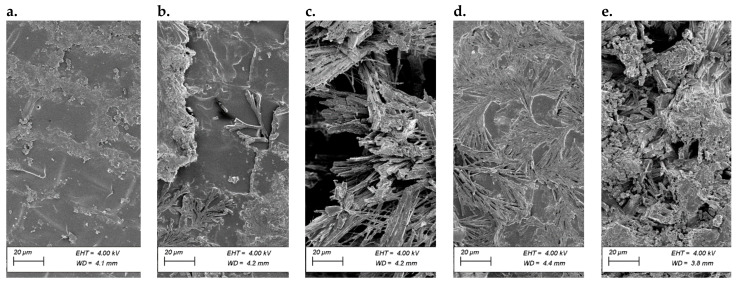
SEM microscopic image of the surface morphology after modification with different concentrations of the AA (**a**) S1—reference sample, (**b**) S5.1 sample, (**c**) S5.2 sample, (**d**) S5.3 sample, (**e**) S5.4 sample; InLENS (S1—Uncoated seeds, S5.1—Seed coating containing a solution of 2% alginate with amino acids (37.6 mL/100 g), micronutrients (37.5 mL/100 g), and NPK, S5.2—Seed coating containing a solution of 2% alginate with amino acids (75.1 mL/100 g), micronutrients (75.1 mL/100 g), and NPK, S5.3—Seed coating containing a solution of 2% alginate with amino acids (112 mL/100 g), micronutrients (112 mL/100 g), and NPK, S5.4—Seed coating containing a solution of 2% alginate with amino acids (150 mL/100 g), micronutrients (150 mL/100 g), and NPK).

SEM microscopic images allowed the observation of surface changes resulting from applying different doses of both micronutrients and AA. Sample S1, the native sample, was used as a reference (Figure 3a). On the surface of this sample, only epidermal cells were visible, including stomata and surrounding guard cells. For the first stage of modification (Figure 3b), the microscopic images clearly showed a coating not present on the entire surface of the sample. This coating was wavy and had an uneven structure. Such morphology indicates the beginning of the crystallization of the coating. Samples numbered S5.2 (Figure 2C) were characterized by a completely invisible natural seed surface. This surface was covered by an uneven layer that was spatially structured and composed of randomly distributed fibrous structures. This surface was very characteristic. Such a strong surface variation was no longer present in other samples. Successive micronutrient concentrations and the AA dosage caused the coating to behave differently. The patterns resembled a ginkgo leaf (Figure 2D), which indicates a specific course of the crystallization front of the coating. Figure 3e shows a surface characterized by flattened and fibrillary areas with a predominance of the latter. There were two types of structures present on the layer: a surface consisting of fine, irregular spherical coagulated particles and a surface composed of columnar-looking crystalline structures previously described as fibrous.

**Figure 4:**
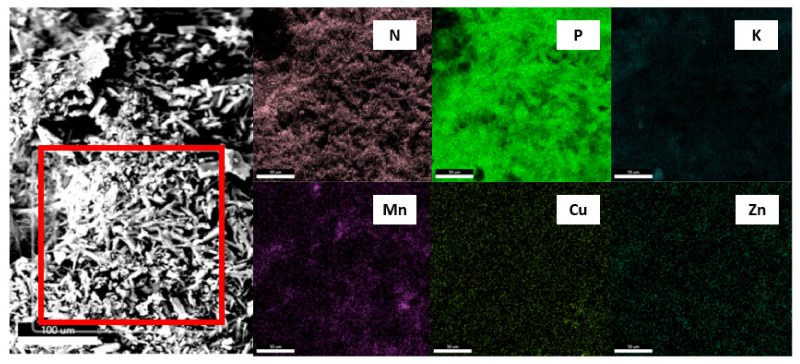
Sample surface mapping with ROI selected (area marked in red). Element map for ROI.

The SEM-EDAX analysis made it possible to compare the percentages of elements in specific microareas of the samples. The elements mapping of the surface revealed the chemical composition of the elements N, P, K, and microelements, i.e., Mn, Cu, and, Zn, on the surface of the seeds (Table 6). A correlation was also found between the thickness of the AA layer and the layer containing the micronutrients. As the thickness of the AA envelope increased, the concentration of micronutrients on the surface decreased, which demonstrates the increasing integrity of the coating. A single layer of AA may not be sufficient to cover the surface completely. The increased presence of manganese in a layer containing micronutrients formed strong fibrous structures (Figure 4). In the case of locally observed very high concentrations of all micronutrients, the surface was very strongly fibrous. The measurement of small micro-areas suggested that the chemical composition of these surfaces was not homogeneous.

##### Composition Analysis

Delivery of nutrients by seed treatment and coating is becoming appreciated in the era of precise approach towards plant nutrition. Seed treatment by soaking or coating has long been a well-known method. Many variables impact the effectiveness of the process, such as soaking time, the concentration of nutrients in the solution, and temperature [29]. Seed coating refers mainly to applying a coating of finely ground components or the application of dissolved solid components without dipping the seeds [30].

Kataki et al. (2008), in their work, demonstrated that the application of seeds enriched with micronutrients increases germination and yield by up to 40%. The effect of coating containing micronutrients, plant growth regulators and amino acids on germination percentage and emergence rate index was also investigated [31]. Polymer L88(R) was used as a thickening agent. Seed coating had a positive effect on the rate of emergence of pee plants [32]. In their study, Freiberg et al. (2017) examined the effect of seed treatment on seed quality during storage. Seeds were coated with a blend of micronutrients (Mn, Mo, Zn) and plant protection agents with a polymer (Colorseed He). No difference was found between the polymer-coated seeds and the untreated seeds. It was observed that the quality of stored seeds was not affected by micronutrients in any way. The lack of adverse effects of the coatings on seed storage and the potential for increased yields with the ability to reduce traditional fertilizer application rate makes this method very prospective [33].

This method can significantly improve the environment. The natural polymer will allow for controlled release of elements so that even high levels of individual nutrients in the coating will not be released too fast into the soil and groundwater. For this reason, the results of macro- and micronutrient composition analyses in enrichment media and coated seeds are favorable (Table 7). Double- or multiple-seed spraying allows plants to receive about 0.1% of each of the tested micronutrients (Cu, Mn, Zn) in an easily accessible form (divalent ions). Mono-ammonium phosphate (MAP) and ammonium nitrate (NH_4_NO_3_) contained in the enrichment media do not pose an environmental hazard (ammonia and NO_x_ emissions) because they are encapsulated in an alginate hydrogel coating that prevents the release of nitrogen compounds into the atmosphere and groundwater [34]. Alkaline hydrolysis of mealworm larvae also provided amino acids as an additional valuable source of nitrogen with high bioavailability (the total nitrogen content of coated cucumber seeds varies in a range of 5%). Nitrogen from amino acids has a stimulatory effect on plants because amino acids can be readily incorporated into their metabolic pathways. Amino acids can enhance the transport and absorption of micronutrients because they are chelators of metal ions [35]. Seed coating medium has many advantages: it is a source of NPK with chelated micronutrients (Cu(II), Zn(II), Mn(II)) with high bioavailability and stimulatory activity.

#### 3.2.2. The Extraction Tests

Extraction tests are usually carried out to assess the bioavailability of nutrients in fertilizers on a laboratory scale. The amount of macro and micronutrients in a form useful for plants is influenced by their genotype, climatic and soil conditions, and the types of agricultural practices [36]. The water and neutral ammonium citrate extraction test results are shown in Table 8. The experiments clearly showed that the macro- and micronutrients contained in seed coats were in plant-available form. Extraction tests simulated nutrient release. The significantly higher desorption of macro- and micronutrients in ammonium citrate than in water is related to the higher ionic strength of the solution. The increased bioavailability of macro- and micronutrients in ammonium citrate reflecting soil solution is highly desirable, as the coated seeds already have access to available forms of nutrients during the first stages of growth [37]. The nutrients are encapsulated in a hydrogel coat that acts as a barrier to them and prevents their immediate transport into the soil. Micronutrients are the crosslinking agent that enables the formation of a coating, and their diffusion from the hydrogel matrix occurs in a controlled manner, as demonstrated in a study by Dawid Skrzypczak et al. (2019) [34]. The proposed method of coating seeds with NPK and micronutrients is safe for the environment due to the low availability of nutrients in the water, protecting from leaching of nutrients, and during rainfall, they will not be lost due to runoff into deeper parts of the soil [37].

A larger-scale implementation of the seed-coating method presented in this paper would allow the nutritional needs of plants (in highly bioavailable forms of macro and microelements) to be satisfied from the germination stage on without the requirement for additional fertilizer applications. This would result in reducing the problem of over-fertilization and its effects on the ecosystem.

#### 3.2.3. Pot Tests

**Figure 5:**
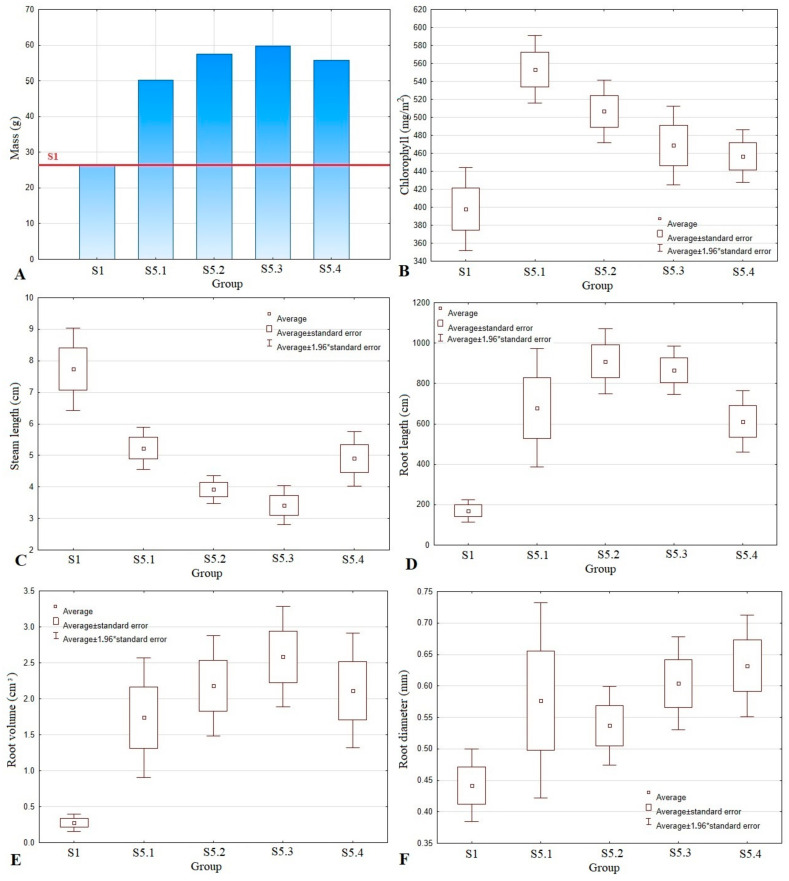
Cucumber sprout growth parameters: fresh mass (**A**), chlorophyll content (**B**), steam length (**C**), root length (**D**), root volume (**E**), and root diameter (**F**) (S1—Uncoated seeds, S5.1—Seed coating containing a solution of 2% alginate with amino acids (37.6 mL/100 g), micronutrients (37.5 mL/100 g), and NPK, S5.2—Seed coating containing a solution of 2% alginate with amino acids (75.1 mL/100 g), micronutrients (75.1 mL/100 g), and NPK, S5.3—Seed coating containing a solution of 2% alginate with amino acids (112 mL/100 g), micronutrients (112 mL/100 g), and NPK, S5.4—Seed coating containing a solution of 2% alginate with amino acids (150 mL/100 g), micronutrients (150 mL/100 g), and NPK).

Pot test plants were subjected to a biometric evaluation of the chlorophyll content, mass, stem length, and root area, diameter, length, and volume. The results were statistically analyzed and are presented in Figure 5 and Table 9.

The longest stem was recorded for the control group—7.74 cm—and this result was statistically significant. The other measured parameter values for the control group were the lowest. Formulation S5.3, where seeds were coated with three layers, produced the highest plant weight, although the differences for other groups with coated seeds were insignificant. The thinnest coating (one layer) had a statistically significant effect on the chlorophyll content, leading to the highest value of 553 mg/m^2^.

Plants that grew from seeds covered with two layers of NPK, micronutrients, and AA immobilized in ALG had the longest roots (almost 9.1 cm), while the highest dose of NPK, micronutrients, and AA introduced in a quadruple coating produced the highest root diameter (1.10 mm).

**Figure 6:**
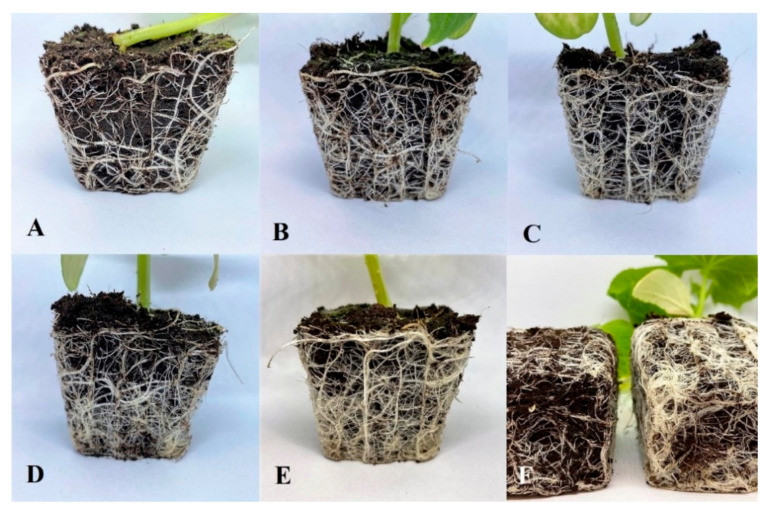
Root ball arrangement in pots for S1 (**A**), S5.1 (**B**), S5.2 (**C**), S5.3 (**D**), S5.4 (**E**), and S1 and S5.4 (**F**) (S1—Uncoated seeds, S5.1—Seed coating containing a solution of 2% alginate with amino acids (37.6 mL/100 g), micronutrients (37.5 mL/100 g), and NPK, S5.2—Seed coating containing a solution of 2% alginate with amino acids (75.1 mL/100 g), micronutrients (75.1 mL/100 g), and NPK, S5.3—Seed coating containing a solution of 2% alginate with amino acids (112 mL/100 g), micronutrients (112 mL/100 g), and NPK, S5.4—Seed coating containing a solution of 2% alginate with amino acids (150 mL/100 g), micronutrients (150 mL/100 g), and NPK).

**Figure 7:**
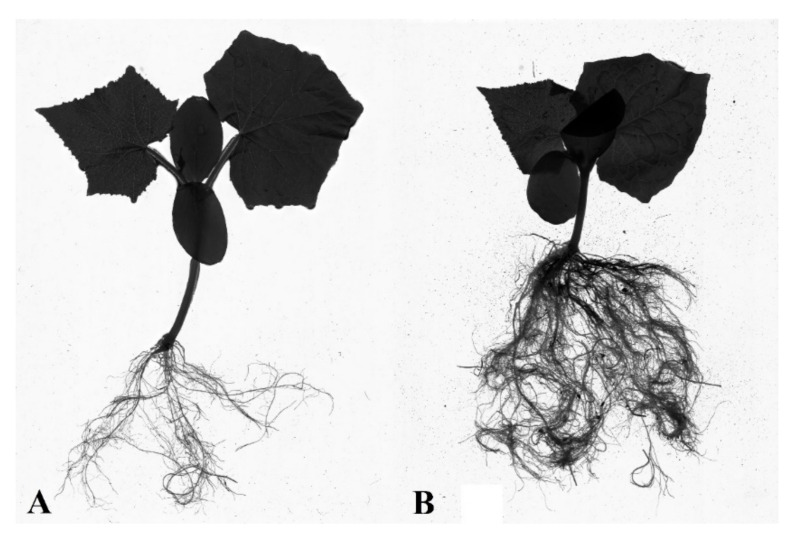
Overground part and root ball of cucumber in pots for S1 (**A**) and S5.3 (**B**). (S1—Uncoated seeds, S5.3—Seed coating containing a solution of 2% alginate with amino acids (112 mL/100 g), micronutrients (112 mL/100 g), and NPK).

Triple seed coating of alginate with NPK, micronutrients, and AA affected the increase in the root area, although these results were statistically significant only against the control group. A similar relation for the S5.3 group was obtained for root volume, with the average value at 2.58 cm^3^. Although it was the highest, it was only statistically significant in relation to S1. The difference in root volume can be seen in Figure 6 and Figure 7. Root system compaction is a highly desirable phenomenon because it increases the uptake of water and nutrients.

The biomass yield was subjected to analysis of the micro- and macronutrient content. This allowed the calculation of the transfer factor (TF, %) of fertilizer nutrients with respect to plant weight, enabling the evaluation of coating efficiency and bioavailability of nutrients released from the coating. Results are presented in Table 10.

As expected, the highest content of micronutrients (Cu, Mn, Zn) was observed at the highest dose of micronutrients, which was introduced together with the coating. Considering the weight of plants in the S5.4 group, which was much higher than the control group, it can be concluded that the highest dose did not cause a phytotoxic effect and thus did not inhibit germination. The highest content of P and K was recorded for group S5.3. An inverse relationship was observed for the TF transfer factor. The lowest dose applied to the coating had the highest values. The lowest mass was also recorded for this group, which also increased the TF factor. The determined transfer of micronutrients to plant biomass shows that micronutrients are still present in the soil and can be taken up by plants (TF is lower than 100%).

Seed coating effectively enhances plant growth at the initial stages, as evidenced by the study and literature data. The coating of wheat seeds with zinc salts (chlorides and sulfate) positively affects germination capacity, stem and root length, plant dry matter, and Zn content in wheat grain. The addition of sulfate is more beneficial to plants because Cl^-^ ions can have an inhibitory effect on plant growth [38]. The positive effect of micronutrients (Zn, B, Mo, Cu, Fe, and Mn) introduced into the maize seed coat along with PGPR bacteria (plant growth-promoting rhizobacteria) was demonstrated in a study by Saadat et al. (2015) [39]. This combination’s efficacy was shown in field trials, with improvements in quantitative and qualitative yield characteristics. Maize was also the subject of Ali et al.’s (2015) research [40]. Seeds coated with zinc and boron tested in field trials yielded about 25% more than the control group. The potential of sodium alginate coating is also known. Primarily, it is used as a carrier for rhizosphere bacteria [41] and AA or plant extracts [42]. Due to its structure, sodium alginate is believed to exhibit controlled-release characteristics, resulting in less environmental loss and longer plant access to the active ingredients introduced with the envelope [43].

## 4. Conclusions

Seed coating is a promising method for precision agriculture for delivering macro- and micronutrients at the early stages of plant growth. The additional coating with amino acids is in line with new trends regarding plant growth biostimulants and nitrogen applied in the form of amino acids.

Experiments highlighted the feasibility of multilayer seed coating containing a biopolymer with macronutrients, a micronutrient solution, and amino acids derived from the hydrolysis of mealworm larvae. Multi-element composition analysis and SEM-EDX confirmed the high concentrations of the nutrients and indicated that they were not uniformly distributed on the surface, which did not have a significant effect on germination. Extraction tests showed reduced leaching of nutrients into the water and the presence of highly bioavailable forms of macro- and micronutrients that are released to stimulated soil solution. Pot tests showed that amino acids present in the outer seed coat stimulated root ball growth. With a more extensive root system, the plant can take up higher doses of nutrients from the soil and consequently the plant growth is stimulated.

The proposed method can be an alternative to fertilizing crops with multicomponent mineral fertilizers at the early stages of plant growth, especially in organic farming systems. Further research is needed to improve the process of seeds coating, with particular emphasis on achieving coating uniformity.

## Figures and Tables

**Table 1:** Composition of seeds coating solutions (ALG—sodium alginate, GA—gum arabic, CMC—carboxymethyl cellulose, AA—amino acid solution).

Sample	Solution	ALG	GA	CMC	AA
		%	%	%	mL/100 g
S1	-	-	-	-	-
S2	NPK + ALG	2	-	-	-
S3	NPK + ALG	4	-	-	-
S4	NPK + CMC + ALG	2	-	1	-
S5	NPK + ALG + AA	2	-	-	37.5
S6	NPK+ GA	-	2	-	-
S7	NPK + GA	-	4	-	-
S8	NPK + GA + AA	-	4	-	37.5

**Table 2:** Compositions of seeds coating solutions (ALG—sodium alginate, M—microelements solution, GA—gum arabic, CMC—carboxymethyl cellulose, AA—amino acid solution).

Sample	Coating Composition	ALG	M	AA
		%	mL/100 g	mL/100 g
S5.1	NPK + ALG + M + B	2	37.5	37.6
S5.2	NPK + ALG + M + B	2	75.0	75.1
S5.3	NPK + ALG + M + B	2	112	112
S5.4	NPK + ALG + M + B	2	150	150

**Table 3:** The concentration of amino acids in the sample based on three technical replicates.

Amino Acid	Concentration (mM)
Serine	31.4 ± 1.5
Proline	48.8 ± 1.5
Glutamate	55.8 ± 1.3
Methionine	2.48 ± 0.58
Aspartate	9.19 ± 0.51
Phenylalanine	2.12 ± 0.41
Isoleucine	4.33 ± 0.31
Tyrosine	9.93 ± 0.29
Threonine	8.49 ± 0.26
Valine	8.11 ± 0.24
Alanine	19.5 ± 0.1
Leucine	10.1 ± 0.7
Glycine	71.2 ± 0.6
Total	281 ± 7

**Table 4:** Germination power of coated seeds (S1—Uncoated seeds, S2—Seed coating containing a solution of 2% alginate with NPK, S3—Seed coating containing a solution of 4% alginate with NPK, S4—Seed coating containing a solution of 2% alginate, 1% carboxymethyl cellulose with NPK, S5—Seed coating containing a solution of 2% alginate with amino acids (37.5 mL/100 g) and NPK, S6—Seed coating containing a 2% solution of gum arabic with NPK, S7—Seed coating containing a solution of 4% gum arabic with NPK, S8—Seed coating containing a solution of 4% gum arabic with amino acids (37.5 mL/100 g) and NPK).

Day	Germination Power							
	%							
	S1	S2	S3	S4	S5	S6	S7	S8
1	44	48	12	56	8	0	0	0
2	96	100	76	92	92	16	32	0
3	100	100	88	96	100	16	32	8

**Table 5:** Effect of thickening agent on growth parameters of cucumber—germination test seeds (S1—Uncoated seeds, S2—Seed coating containing a solution of 2% alginate with NPK, S3—Seed coating containing a solution of 4% alginate with NPK, S4—Seed coating containing a solution of 2% alginate, 1% carboxymethyl cellulose with NPK, S5—Seed coating containing a solution of 2% alginate with amino acids (37.5 mL/100 g) and NPK).

Group	Solution				Chlorophyll	Stem Length	Root Area	Root Length	Root Volume
	ALG	GA	CMC	AA					
	%	%	%	mL/100 g	mg/m^2^	mm	cm^2^	cm	cm^3^
S1	-	-	-	-	646 ± 77 ^a,b^	16.8 ± 2.2 ^a,b,c^	6.74 ± 1.73 ^a^	37.6 ± 9.6	0.100 ± 0.030 ^a^
S2	2	-	-	-	552 ± 93 ^a,c^	13.9 ± 1.2 ^a,d,e^	7.07 ± 2.40 ^b^	40.9 ± 16.5	0.100 ± 0.030 ^b^
S3	4	-	-	-	540 ± 87 ^b,d^	11.2 ± 1.6 ^b,d,f,g^	6.46 ± 2.58 ^c^	47.6 ± 15.8 ^a^	0.0700 ± 0.0400 ^c,d^
S4	2	-	1	-	673 ± 42 ^c,d,e^	14.6 ± 1.8 ^c,f,h^	6.51 ± 1.85 ^d^	33.7 ± 9.8	0.100 ± 0.030 ^c,e^
S5	2	-	-	37.5	577 ± 57 ^e^	16.8 ± 1.7 ^d,g,h^	9.09 ± 2.07 ^a,b,c,d^	46.6 ± 11.9 ^a^	0.140 ± 0.040 ^a,b,d,e^

^a–h^—results marked with the same letter differ significantly statistically—Tukey’s test (*p* < 0.05, vertical comparison).

**Table 6:** Chemical composition of the microareas of the surface of the seeds, obtained by the energy dispersive X-ray analysis (S1—Uncoated seeds, S5.1—Seed coating containing a solution of 2% alginate with amino acids (37.6 mL/100 g), micronutrients (37.5 mL/100 g), and NPK, S5.2—Seed coating containing a solution of 2% alginate with amino acids (75.1 mL/100 g), micronutrients (75.1 mL/100 g), and NPK, S5.3—Seed coating containing a solution of 2% alginate with amino acids (112 mL/100 g), micronutrients (112 mL/100 g), and NPK, S5.4—Seed coating containing a solution of 2% alginate with amino acids (150 mL/100 g), micronutrients (150 mL/100 g), and NPK).

Sample	N	P	K	Mn	Cu	Zn
	%					
S1	80.9 ± 2.1 ^a,b,c,d^	8.63 ± 1.24 ^a,b,c^	7.40 ± 2.33 ^a,b,c,d^	N/A	N/A	N/A
S5.1	23.2 ± 10.0 ^a,e,f^	28.87 ± 14.4	44.5 ± 6.1 ^a^	2.43 ± 1.93	0.300 ± 0.100	0.567 ± 0.503
S5.2	2.57 ± 3.09 ^b,e^	43.3 ± 8.12 ^a^	45.9 ± 2.5 ^b^	2.43 ± 0.70	4.40 ± 0.60	1.50 ± 1.21
S5.3	8.06 ± 2.68 ^c,f^	36.5 ± 4.3 ^b^	51.0 ± 5.5 ^c^	1.93 ± 0.70	1.36 ± 0.57	1.20 ± 0.62
S5.4	12.4 ± 2.0 ^d^	44.0 ± 1.7 ^c^	38.5 ± 3.8 ^d^	3.27 ± 2.64	1.10 ± 0.66	0.833 ± 0.462

^a–f^—results marked with the same letter differ significantly statistically—Tukey’s test (*p* < 0.05, vertical comparison).

**Table 7:** Macro and micronutrient levels in enrichment media and coated seeds (M—micronutrient solution, ALG—alginate, S1—Uncoated seeds, S5.1—Seed coating containing a solution of 2% alginate with amino acids (37.6 mL/100 g), micronutrients (37.5 mL/100 g), and NPK, S5.2—Seed coating containing a solution of 2% alginate with amino acids (75.1 mL/100 g), micronutrients (75.1 mL/100 g), and NPK, S5.3—Seed coating containing a solution of 2% alginate with amino acids (112 mL/100 g), micronutrients (112 mL/100 g), and NPK, S5.4—Seed coating containing a solution of 2% alginate with amino acids (150 mL/100 g), micronutrients (150 mL/100 g), and NPK).

Materials		N	P	K	Cu	Mn	Zn
		%	%	%	mg/L	mg/L	mg/L
Enrichment solutions	AA	2.58 ± 0.39	3.65 ± 0.55	5.73 ± 0.86	2.49 ± 0.37	1.67 ± 0.25	9.66 ± 1.45
	ALG+NPK solution	1.02 ± 0.15	1.45 ± 0.22	2.14 ± 0.32	0.391 ± 0.059	14.0 ± 2.1	19.0 ± 2.9
	M	-	-	-	6224 ± 934	5104 ± 766	5072 ± 761
Cucumber seeds	S1	4.14 ± 0.62	1.08 ± 0.16	0.722 ± 0.108	21.2 ± 3.2	38.6 ± 5.8	83.9 ± 12.6
	S5.1	5.21 ± 0.78	2.83 ± 0.42	3.52 ± 0.53	499 ± 74	482 ± 72	524 ± 79
	S5.2	5.29 ± 0.79	2.87 ± 0.43	3.58 ± 0.54	1128 ± 169	957 ± 144	1003 ± 151
	S5.3	5.01 ± 0.75	2.89 ± 0.43	3.53 ± 0.53	1579 ± 237	1370 ± 206	1463 ± 219
	S5.4	5.01 ± 0.75	3.07 ± 0.46	3.94 ± 0.59	1833 ± 275	1421 ± 213	1571 ± 236

**Table 8:** Evaluation of leachability of elements in the coating—water and neutral ammonium citrate extraction (S1—Uncoated seeds, S5.1—Seed coating containing a solution of 2% alginate with amino acids (37.6 mL/100 g), micronutrients (37.5 mL/100 g), and NPK, S5.2—Seed coating containing a solution of 2% alginate with amino acids (75.1 mL/100 g), micronutrients (75.1 mL/100 g), and NPK, S5.3—Seed coating containing a solution of 2% alginate with amino acids (112 mL/100 g), micronutrients (112 mL/100 g), and NPK, S5.4—Seed coating containing a solution of 2% alginate with amino acids (150 mL/100 g), micronutrients (150 mL/100 g), and NPK).

Extractor	Materials	P	K	Cu	Mn	Zn
		%				
Water extraction	S1	0.00	0.685	1.96	3.25	2.16
	S5.1	5.82	7.51	14.1	12.4	5.33
	S5.2	7.54	9.48	7.10	9.64	5.26
	S5.3	5.50	7.07	4.48	9.01	6.89
	S5.4	7.75	9.24	5.46	9.48	6.71
Neutral ammonium citrate extraction	S1	7.60	9.11	56.9	32.2	19.4
	S5.1	69.8	68.4	100	100	100
	S5.2	60.5	59.6	100	100	92.3
	S5.3	67.5	65.9	100	100	86.0
	S5.4	66.2	57.4	100	82.5	67.1

**Table 9:** Effect of coating thickness with micronutrients and AAt on growth parameters of cucumber—pot tests) (S1—Uncoated seeds, S5.1—Seed coating containing a solution of 2% alginate with amino acids (37.6 mL/100 g), micronutrients (37.5 mL/100 g), and NPK, S5.2—Seed coating containing a solution of 2% alginate with amino acids (75.1 mL/100 g), micronutrients (75.1 mL/100 g), and NPK, S5.3—Seed coating containing a solution of 2% alginate with amino acids (112 mL/100 g), micronutrients (112 mL/100 g), and NPK, S5.4—Seed coating containing a solution of 2% alginate with amino acids (150 mL/100 g), micronutrients (150 mL/100 g), and NPK).

Group	Chlorophyll	SteamLength	Root Area	Root Diameter	Root Length	Root Volume
	mg/m^2^	cm	cm^2^	mm	mm	cm^3^
S1	398 ± 74 ^a,b^	7.74 ± 2.21 ^a,b,c,d^	23.5 ± 15.0 ^a,b,c,d^	0.440 ± 0.11	170 ± 16 ^a,b,c,d^	0.280 ± 0.210 ^a,b,c,d^
S5.1	553 ± 6 ^a,c,d^	5.23 ± 1.08 ^a,e^	117 ± 77 ^a^	0.580 ± 0.240	679 ± 48 ^a^	1.74 ± 0.27 ^a^
S5.2	507 ± 5 ^b^	3.92 ± 0.71 ^b^	156 ± 58 ^b^	0.540 ± 0.100	909 ± 60 ^b,e^	2.18 ± 0.12 ^b^
S5.3	469 ± 67 ^c^	3.42 ± 0.95 ^c,e^	165 ± 50 ^c^	0.600 ± 0.110	865 ± 18 ^c^	2.58 ± 0.07 ^c^
S5.4	457 ± 50 ^d^	4.90 ± 1.46 ^d^	115 ± 67 ^d^	1.10 ± 0.14	557 ± 29 ^d,e^	2.45 ± 0.65 ^d^

^a–e^—results marked with the same letter differ significantly statistically—Tukey’s test (*p* < 0.05, vertical comparison).

**Table 10:** Macro- and micronutrient content of plants and effect of coating on bioavailability (TF) of micronutrients (S1—Uncoated seeds, S5.1—Seed coating containing a solution of 2% alginate with amino acids (37.6 mL/100 g), micronutrients (37.5 mL/100 g), and NPK, S5.2—Seed coating containing a solution of 2% alginate with amino acids (75.1 mL/100 g), micronutrients (75.1 mL/100 g), and NPK, S5.3—Seed coating containing a solution of 2% alginate with amino acids (112 mL/100 g), micronutrients (112 mL/100 g), and NPK, S5.4—Seed coating containing a solution of 2% alginate with amino acids (150 mL/100 g), micronutrients (150 mL/100 g), and NPK).

Group	Content					TF		
	mg/kg					%		
	Cu	Mn	Zn	P	K	Cu	Mn	Zn
S1	7.88 ± 1.18	44.3 ± 6.7	59.1 ± 8.9	6835 ± 1025	34838 ± 5226	-	-	-
S5.1	8.92 ± 1.34	46.7 ± 7.0	83.7 ± 12.6	7860 ± 1179	45024 ± 6754	10.3 ± 1.6	58.3 ± 8.8	82.3 ± 12.4
S5.2	11.4 ± 1.7	47.5 ± 7.1	81.8 ± 12.3	8939 ± 1341	46731 ± 7010	6.71 ± 1.01	32.2 ± 4.8	55.6 ± 8.3
S5.3	11.0 ± 1.7	56.3 ± 8.5	106 ± 16	9089 ± 1364	47231 ± 7085	8.20 ± 1.23	27.9 ± 4.2	79.4 ± 11.9
S5.4	13.9 ± 2.1	72.5 ± 10.9	154 ± 23	8834 ± 1325	4589 ± 688	5.52 ± 0.83	26.5 ± 4.0	77.5 ± 11.6

## Data Availability

Data is contained within the article.

## Abbreviations

AA	Amino acids
Ala	Alanine
ALG	Sodium alginate
Asp	Aspartate
CMC	Carboxymethyl cellulose
GA	Gum arabic
Glu	Glutamate
Gly	Glycine
ICP-OES	Inductively coupled plasma optical emission spectrometry
Ile	Isoleucine
Leu	Leucine
M	Micronutrient solution
MAP	Mono-ammonium phosphate
Met	Methionine
Phe	Phenylalanine
Pro	Proline
S1	Uncoated seeds
S2	Seed coating containing a solution of 2% alginate with NPK
S3	Seed coating containing a solution of 4% alginate with NPK
S4	Seed coating containing a solution of 2% alginate, 1% carboxymethyl cellulose with NPK
S5	Seed coating containing a solution of 2% alginate with amino acids (37.5 mL/100 g) and NPK
S5.1	Seed coating containing a solution of 2% alginate with amino acids (37.6 mL/100 g), micronutrients (37.5 mL/100 g) and NPK
S5.2	Seed coating containing a solution of 2% alginate with amino acids (75.1 mL/100 g), micronutrients (75.1 mL/100 g) and NPK
S5.3	Seed coating containing a solution of 2% alginate with amino acids (112 mL/100 g), micronutrients (112 mL/100 g) and NPK
S5.4	Seed coating containing a solution of 2% alginate with amino acids (150 mL/100 g), micronutrients (150 mL/100 g) and NPK
S6	Seed coating containing a 2% solution of gum arabic with NPK
S7	Seed coating containing a solution of 4% gum arabic with NPK
S8	Seed coating containing a solution of 4% gum arabic with amino acids (37.5 mL/100 g) and NPK
SEM-EDX	Scanning electron microscopy with energy-dispersive X-ray spectroscopy
Ser	Serine
Thr	Threonine
Tyr	Tyrosine
Val	Valine
